# Rspo2 exacerbates rheumatoid arthritis by targeting aggressive phenotype of fibroblast-like synoviocytes and disrupting chondrocyte homeostasis via Wnt/β-catenin pathway

**DOI:** 10.1186/s13075-023-03198-1

**Published:** 2023-11-09

**Authors:** Dong Guo, Haoyan Pan, Xueying Lu, Zhong Chen, Laixi Zhou, Shuxin Chen, Jin Huang, Xinzhi Liang, Zhisheng Xiao, Hua Zeng, Yan Shao, Weizhong Qi, Denghui Xie, Chuangxin Lin

**Affiliations:** 1 Department of Orthopedic Surgery, Center for Orthopedic Surgery, The Third Affiliated Hospital of Southern Medical University, Guangzhou, 510630 People’s Republic of China; 2 Guangdong Provincial Key Laboratory of Bone and Joint Degeneration Diseases, Guangzhou, 510630 People’s Republic of China; 3 Shenzhen Hospital of Beijing University of Chinese Medicine (Longgang), Shenzhen, 518100 People’s Republic of China; 4 Department of Orthopedics, Sun Yat-Sen Memorial Hospital, Sun Yat-Sen University, Guangzhou, 510120 People’s Republic of China; 5 Department of Orthopedic Surgery, Shantou Central Hospital, Affiliated Shantou Hospital of Sun Yat-Sen University, Shantou, 515031 People’s Republic of China

**Keywords:** Rspo2, Fibroblast-like synoviocyte, Chondrocyte, Synovitis, Wnt/ β-catenin, Rheumatoid arthritis

## Abstract

**Background:**

The aggressive phenotype of fibroblast-like synoviocytes (FLS) has been identified as a contributing factor to the exacerbation of rheumatoid arthritis (RA) through the promotion of synovitis and cartilage damage. Regrettably, there is currently no effective therapeutic intervention available to address this issue. Recent research has shed light on the crucial regulatory role of R-spondin-2 (Rspo2) in cellular proliferation, cartilage degradation, and tumorigenesis. However, the specific impact of Rspo2 on RA remains poorly understood. We aim to investigate the function and mechanism of Rspo2 in regulating the aggressive phenotype of FLS and maintaining chondrocyte homeostasis in the context of RA.

**Methods:**

The expression of Rspo2 in knee joint synovium and cartilage were detected in RA mice with antigen-induced arthritis (AIA) and RA patients. Recombinant mouse Rspo2 (rmRspo2), Rspo2 neutralizing antibody (Rspo2-NAb), and recombinant mouse DKK1 (rmDKK1, a potent inhibitor of Wnt signaling pathway) were used to explore the role and mechanism of Rspo2 in the progression of RA, specifically in relation to the aggressive phenotype of FLS and chondrocyte homeostasis, both in vivo and in vitro.

**Results:**

We indicated that Rspo2 expression was upregulated both in synovium and articular cartilage as RA progressed in RA mice and RA patients. Increased Rspo2 upregulated the expression of leucine-rich repeat-containing G-protein-coupled receptor 5 (LGR5), as the ligand for Rspo2, and β-catenin in FLS and chondrocytes. Subsequent investigations revealed that intra-articular administration of rmRspo2 caused striking progressive synovitis and articular cartilage destruction to exacerbate RA progress in mice. Conversely, neutralization of Rspo2 or inhibition of the Wnt/β-catenin pathway effectively alleviated experimental RA development. Moreover, Rspo2 facilitated FLS aggressive phenotype and disrupted chondrocyte homeostasis primarily through activating Wnt/β-catenin pathway, which were effectively alleviated by Rspo2-NAb or rmDKK1.

**Conclusions:**

Our data confirmed a critical role of Rspo2 in enhancing the aggressive phenotype of FLS and disrupting chondrocyte homeostasis through the Wnt/β-catenin pathway in the context of RA. Furthermore, the results indicated that intra-articular administration of Rspo2 neutralizing antibody or recombinant DKK1 might represent a promising therapeutic strategy for the treatment of RA.

**Supplementary Information:**

The online version contains supplementary material available at 10.1186/s13075-023-03198-1.

## Background

Rheumatoid arthritis (RA) is a chronic systemic autoimmune disease whose clinical manifestations are mainly synovial inflammation and joint damage [1]. During the development of RA, the articular synovium transforms into hyperplastic infiltrating tissue, causing cartilage and bone destruction with a decreased quality of life [2]. There is no treatment for RA, and the available therapeutic regimens can only partially alleviate symptoms and improve survival [3]. Despite major advances and improved efficacy of targeted therapy, a significant proportion of those affected still suffer from persistent inflammation and progressive disability [4]. Fibroblast-like synoviocytes (FLS) have also been implicated in the pathogenesis of RA as a stromal component of the synovial intima lining. RA FLS display aggressive phenotypes and produce pathogenic cytokines which cause the onset and development of RA [5]. Therefore, targeting RA FLS aggressive phenotype may be a promising approach to attenuate synovial inflammation and alleviate cartilage damage in RA.

The synovium consists of two layers, an intimal lining layer and a sublining layer. FLS mainly reside in the former. The intimal lining is a thin porous layer that separates the synovial fluid from the sublining [6]. The composition of the synovial fluid and extracellular matrix (ECM) are regulated by healthy FLS. However, activated RA FLS develop distinct aggressive phenotype which play a vital role in RA pathogenesis and progression [7]. RA FLS cannot be considered as merely passive responders to the inflammatory milieu, since their aggressive phenotypes are independent of inflammatory milieu, despite the fact that inflammatory cytokines are crucial regulators of stromal cells [8]. Firestein et al. previously reported that FLS from RA patients exhibited autonomous pathogenic properties which remained months of tissue culture or implantation into mice [9]. However, the molecular mechanisms underlying the aggressive phenotype of FLS are still unclear.

R-spondins are a family of Wnt activators. In the R-spondin family, there are four secreted proteins (Rspo1–4) sharing 40–60% overall sequence homology and containing two furin-like cysteine-rich domains [10, 11]. They were initially discovered as Wnt agonists owing to their strong potentiation of canonical Wnt/β-catenin signaling [12]. Recently, R-spondins had been identified as ligands of the leucine-rich repeat-containing G-protein-coupled receptors (LGRs), including LGR4, 5, and 6 [13]. Numerous studies have examined the important role of R-spondins in various cellular and biological processes, such as cell proliferation, myogenic differentiation, embryonic development, bone formation, and tumorigenesis [14–16]. Research on Rspo2 had accumulated in recent years, particularly regarding its role in activating Wnt signaling [17]. However, the specific effect of Rspo2 in RA remains poorly understood. Compelling evidence has revealed that aberrant activation of Wnt/β-catenin signaling aggravated OA progression by promoting inflammatory cartilage damage in chondrocytes [18]. The dysregulation of Wnt signaling is regarded to be involved in the pathogenesis of various autoimmune diseases, such as RA, systemic lupus erythematosus, systemic sclerosis, ankylosing spondylitis, and psoriasis [19]. Taken together, these findings suggest that Rspo2 may play vital roles in the pathogenesis of RA.

In this study, we investigated the functions of Rspo2 in the onset and development of RA. We found that Rspo2 is highly expressed in the synovium and cartilage of knee joint in patients with RA and mice with antigen-induced arthritis (AIA). The increased Rspo2 exacerbates synovial inflammation and cartilage damage to aggravate the progression of experimental RA by upregulating LGR5 and β-catenin expression. Downregulation of Rspo2 or suppression of the Wnt/β-catenin signaling pathway significantly attenuates RA progression. Rspo2 may therefore be a promising novel therapeutic target for the treatment of RA.

## Methods

### Human samples

Synovium and cartilage from 8 RA patients who underwent a total knee replacement surgery were used in the RA group. Synovium from 8 patients with an ACL injury who had no history of arthritis was used in the control group. Cartilage from the lateral tibial plateau with less cartilage damage of 8 OA patients who underwent a total knee replacement surgery was used in the control group. All human synovium and cartilage tissues were collected from Shantou Central Hospital (Shantou, China). The use of clinical sample for scientific research and written informed consent were obtained from all participants. The Ethics Committee of Shantou Central Hospital approved the study.

### Animals

We purchased sixty 8-week-old male C57BL/6 J mice from Experimental Animal Centre of Southern Medical University (Guangzhou, China). To explore the effect of Rspo2 in RA mouse model, sixty 8-week-old male C57BL/6 J mice were randomly divided into five groups: An intra-articular phosphate-buffered saline (PBS) was administered as the sham group (*n* = 12); an intra-articular 5 μL PBS (vehicle) was administered post modeling antigen-induced arthritis (AIA) as the RA + vehicle group (*n* = 12); Rspo2 group an intra-articular injected with 1 ng/g recombinant mouse Rspo2 (rmRspo2, R&D systems, Minneapolis, MN, USA, #6946-RS) post AIA modeling (*n* = 12); Rspo2 neutralizing antibody (Rspo2-NAb) group with intra-articular injection of 10 ng/g Rspo2-NAb (R&D systems, Minneapolis, MN, USA, #773029) post AIA modeling (*n* = 12); DKK1 group administered with 50 ng/g recombinant mouse DKK1 (rmDKK1, ABclonal Technology Co., Ltd., Wuhan, China, #RP01350LQ) post AIA modeling (*n* = 12). After AIA modeling, the mice (6 mice per time point) were sacrificed either 4 or 8 weeks later. The animals in this study were provided with a consistent diet and kept in cages free of pathogens, with five mice per cage. The temperature and humidity in their living environment remained constant throughout the study. Additionally, their circadian rhythm was maintained at a 12-h cycle. Euthanasia was carried out through the administration of an overdose of ketamine/xylazine anesthesia, followed by cervical dislocation. All animal experiments were conducted with the approval of the Shantou Central Hospital Committee Animal Care and Use Committee and in accordance with their guidelines and regulations.

### Antigen-induced arthritis (AIA)

The experimental model of antigen-induced arthritis (AIA) in mice was conducted according to previously established methods [20]. In brief, an injection of methylated bovine serum albumin (mBSA; Sigma-Aldrich, USA) in 50 mL phosphate-buffered saline (PBS) that was emulsified in 50 mL Freund’s adjuvant (CFA; Sigma-Aldrich, USA) was given subcutaneously to 12-week-old male C57BL/6 J mice on day 0. The mice were injected with 10μL physiological saline containing 10 μg mBSA at the right knee joint after 2 weeks. Sham mice were intra-articularly injected with PBS. The mice were administrated with vehicle, rmRspo2, Rspo2-NAb, or rmDKK1 twice per week on day 3 after modeling AIA.

### Histological analysis

The right knee joints were fixed in 4% paraformaldehyde for 24 h at 4 °C, followed by decalcification with 0.5 M EDTA for 21 days and embedding in paraffin. Safranin O/Fast Green and hematoxylin–eosin (HE) staining were performed on 4-μm-thick sections of the samples. Synovium tissues were graded based on three synovial membrane features (synovial lining cell layer, stroma cell density, and inflammatory infiltrate) using HE-stained slides. A score of 0 to 3 represents no change (score: 0), slight change (score: 1), moderate change (score: 2), and strong change (score: 3) [20]. The Osteoarthritis Research Society International (OARSI) scoring system was used to grade cartilage degeneration in Safranin O/Fast Green stained sections. Using a 0–6 subjective scoring system, we evaluated each joint quadrant as follows: medial femoral condyle (MFC), medial tibial plateau (MTP), lateral femoral condyle (LFC), and lateral tibial plateau (LTP). A score of 0 to 6 represents normal cartilage (score: 0), loss of proteoglycan with an intact surface (score: 0.5), superficial fibrillation without loss of cartilage (score: 1), vertical clefts and loss of surface lamina (score: 2), vertical clefts/erosion to the calcified layer lesion over 1–25% of the quadrant width (score: 3), lesion reaches the calcified cartilage over 25–50% of the quadrant width (score: 4), lesion reaches the calcified cartilage over 50–75% of the quadrant width (score: 5), lesion reaches the calcified cartilage over > 75% of the quadrant width (score: 6). For statistics, we analyzed the average score of each section based on two blinded, independent graders.

### Cell preparation

Fibroblast-like synoviocytes (FLS) were obtained from ATCC. As reported previously, primary chondrocytes were isolated from the newborn mouse tibia cartilage [20]. FLS were treated with 10 ng/ml recombinant human Rspo2 (rhRspo2, R&D systems, Minneapolis, MN, USA, #3266-RS) or 10 ng/ml recombinant human LGR5 (rhLGR5, MedChemExpress, Shanghai, China), with or without 100 ng/ml Rspo2-NAb (R&D systems, Minneapolis, MN, USA, #AF3266) or 500 ng/ml recombinant human DKK1 (rhDKK1, ABclonal Technology Co., Ltd., Wuhan, China, #RP01343) for 24 h to examine phenotype alterations. Primary chondrocytes were treated with 10 ng/ml rmRspo2, with or without 100 ng/ml Rspo2-NAb or 500 ng/ml rmDKK1 for 24 h to examine phenotype alterations.

### Proliferation assay

150,000 FLS were plated per well in a 12-well plate. After 24 h treated with rhRspo2, with or without Rspo2-NAb or rhDKK1, the cells were incubated with 100 μg/mL BrdU (MedChemExpress, Shanghai, China) for 2 h. Three random sights were averaged for the quantification of BrdU-positive cells. A fluorescence microscope (Olympus) was used to obtain the images.

### Migration assay

Transwell inserts (Corning #3422, Sunnyvale, CA, USA) were seeded 250 μL of FLS suspension (50,000 cells per well) in high glucose DMEM without FBS (Gibco, Gaithersburg, MD, USA). Inserts were then placed on 24-well plates filled with 750 μL high glucose DMEM with 10% FBS. After migrating to the lower side of the insert, cells were fixed with methanol and stained with 1% crystal violet for 10 min. Three representative high-power fields of each insert were counted under a microscope (Olympus).

### Invasion assay

Transwell inserts (Corning #3422, Sunnyvale, CA, USA) were seeded 250 μL of FLS suspension (200,000 cells per well) in high glucose DMEM without FBS (Gibco, Gaithersburg, MD, USA). 16% Matrigel (356231; BD Biosciences, San Diego, CA, USA) high glucose DMEM without FBS was used to coat the inserts. After 5 h, inserts were then placed on a 24-well plate filled with 750 μL high glucose DMEM with 20% FBS. After invading to the lower side of the insert, cells were fixed with methanol and stained with 1% crystal violet for 10 min. Three representative high-power fields of each insert were counted under a microscope (Olympus).

### Scratch assay

1,000,000 FLS were plated per well in a 12-well plate. Wounds were scratched in each well using a 200-μl sterile pipette tip after the cells had grown to 100% confluence. The cells were washed with PBS to remove cell debris and subsequently cultured in a basal high glucose DMEM with 10% FBS for 24 h. FLS migration and proliferation across the wound margins were captured using the inverted microscope (Zeiss) and measured using ImageJ software.

### Immunohistochemistry and immunofluorescence

The slides were prepared according to our previous publication [20]. Immunohistochemical staining was performed on sections after blocking with 1% goat serum for an hour at 37 °C. The following primary antibodies were incubated overnight at 4 °C: Rspo2 (1:100 for IHC, 11781–1-AP, Proteintech, Wuhan, China), Ki67 (1:100 for IHC, ab15580, Abcam, Cambridge, UK), ACAN (1:100 for IHC, A11691, Abclonal, Wuhan, China), and MMP13 (1:100 for IHC, 18165–1-AP, Proteintech, Wuhan, China). The sections were incubated with species-matched HRP-conjugated secondary antibodies (1:200 for IHC, Jackson ImmunoResearch Laboratories, West Grove, PA, USA) at room temperature for an hour. Then, substrate chromogen was visualized by diaminobenzidine (DAB; ZSGB-Bio, Beijing, China), and counterstained with hematoxylin. For immunofluorescence, the sections were blocked with 5% BSA containing 0.1% Triton X-100 at room temperature for an hour and then incubated overnight at 4 °C with the following primary antibodies: LGR5 (1:100 for IF, TA503316, OriGene, Rockville, USA), β-catenin (1:100 for IF, ab32572, Abcam, Cambridge, UK), vimentin (1:100 for IF, sc-6260, Santa Cruz, Dallas, USA), MMP3 (1:100 for IHC, 17873–1-AP, Proteintech, Wuhan, China), and Col2a1 (1:100 for IF, ab34712, Abcam, Cambridge, UK). After washing with PBS containing 0.05% Tween-20, the sections were incubated with species-matched Alexa 488 or Alexa 594 fluorescent secondary antibodies (Life Technologies, Carlsbad, CA, USA) at room temperature for an hour, and then counterstained with DAPI (Thermo Fisher Scientific, Waltham, MA, USA). The images were captured by using a fluorescence microscope (Olympus).

### Western blot analysis

Cells and the synovial tissue were immediately lysed with RIPA lysis buffer (FD009, Fudebio, Hangzhou, China) freshly containing phosphatase and protease inhibitors (FD1002 and FD1001, Fudebio, Hangzhou, China) for 10 min on ice. The content of total protein was separated by 10–12% sodium dodecyl sulfate–polyacrylamide gel electrophoresis and transferred to polyvinylidene difluoride (PVDF) membranes (ISEQ00010, Merck Millipore, Darmstadt, Germany). Membranes were blocked with 5% skimmed milk in TBS containing 0.1% Tween-20 (TBST) for an hour at room temperature and incubated with the primary antibodies overnight at 4 °C. After washing with TBST, PVDF membranes were incubated with species-matched HRP-conjugated secondary antibodies (1:5000, Jackson ImmunoResearch Laboratories, West Grove, PA, USA) for an hour at room temperature. Immunoreactive protein bands were visualized with FDbio-Dura ECL Kit (FD8020, Fudebio, Hangzhou, China). Antibodies used for western blotting were as follows: rabbit anti-Rspo2 (1:1000, 11781–1-AP, Proteintech, Wuhan, China), mouse anti-LGR5 (1:2000, TA503316, OriGene, Rockville, USA), rabbit anti-β-catenin (1:1000, ab32572, Abcam, Cambridge, UK), rabbit anti-Col2a1 (1:1000, ab34712, Abcam, Cambridge, UK), rabbit anti-ACAN (1:1000, 13,880–1-AP, Proteintech, Wuhan, China), rabbit anti-SOX9 (1:1000, ab185966, Abcam, Cambridge, UK), and rabbit anti-MMP13 (1:1000, 18165–1-AP, Proteintech, Wuhan, China).

### Quantitative real-time PCR

Total RNA was extracted from cells using TRIzol reagent (Invitrogen, Thermo Fisher Scientific, Waltham, MA, USA) and reverse transcribed using reverse transcription reagents (Vazyme Biotech Co. Ltd, Nanjing, China). cDNA was quantitatively analyzed by using Real-Time PCR Mix (Vazyme Biotech Co. Ltd) on a light cycler (Roche, Basel, Switzerland) with the following primers: human LGR5 Forward 5′-CTC CCA GGT CTG GTG TGT TG-3′, Reverse 5′-GAG GTC TAG GTA GGA GGT GAA G-3′; human β-catenin Forward 5′-AAA GCG GCT GTT AGT CAC TGG-3′, Reverse 5′-CGA GTC ATT GCA TAC TGT CCA T-3′; human TNF-α Forward 5′-CCT CTC TCT AAT CAG CCC TCT G-3′, Reverse 5′-GAG GAC CTG GGA GTA GAT GAG-3′; human IL-1β Forward 5′-ATG ATG GCT TAT TAC AGT GGC AA-3′, Reverse 5′-GTC GGA GAT TCG TAG CTG GA-3′; human IL-6 Forward 5′-ACT CAC CTC TTC AGA ACG AAT TG-3′, Reverse 5′-CCA TCT TTG GAA GGT TCA GGT TG-3′; human β-actin Forward 5′-ACT CAC CTC TTC AGA ACG AAT TG-3′, Reverse 5′-CCA TCT TTG GAA GGT TCA GGT TG-3′; mouse LGR5 Forward 5′-GGA CCA GAT GCG ATA CCG C-3′, Reverse 5′-CAG AGG CGA TGT AGG AGA CTG-3′; mouse β-catenin Forward 5′-ATG GAG CCG GAC AGA AAA GC-3′, Reverse 5′-TGG GAG GTG TCA ACA TCT TCT T-3′; mouse β-actin Forward 5′-GTG ACG TTG ACA TCC GTA AAG A-3′, Reverse 5′-GCC GGA CTC ATC GTA CTC C-3′.

### Enzyme-linked immunosorbent assay (ELISA)

Mouse Rspo2 ELISA Kits (Shanghai Enzyme-linked Biotechnology Co., Ltd., Shanghai, China, #ml037989) were used to analyze the level of Rspo2 in the serum of C57BL/6 J mice. Following the manufacturer’s instructions, the ELISA was performed.

### Small interfering (siRNA) transfection

Twenty μmol/ml siRNA-LGR5 (Tsingke Biological Technology, Wuhan, China) or the corresponding negative control were transfected into FLS using 2.5μL/mL Lipofectamine 3000 (Thermo Fisher Scientific, Waltham, MA, USA) for 48 h, as per the manufacturer’s instructions. Subsequently, the cells were processed with TRIzol reagent for RNA analysis.

SiRNA1-LGR5: 5′-GGA UGA CAA UGC GUU AAC A-3′ (sense), 5′-UGU UAA CGC AUU GUC AUC C-3′ (antisense); SiRNA2-LGR5: 5′-GCU UGG UAG UUC UAC AUC U-3′ (sense), 5′-AGA UGU AGA ACU ACC AAG C-3′ (antisense); SiRNA3-LGR5: 5′-GAC ACU CUC CAA CCU UAA A-3′ (sense), 5′-UUU AAG GUU GGA GAG UGU C-3′ (antisense); SiRNA negative control: 5′-GUA UGA CAA CAG CCU CAA GTT-3′ (sense), 5′-CUU GAG GCU GUU GUC AUA CTT-3′ (antisense).

### Statistical analysis

All experimental analyses were performed in triplicate and were observed by two independent observers. Student’s *t*-tests were used to analyze differences between two groups, whereas one-way analysis of variance (ANOVA) with Tukey’s multiple comparison test was used for analyzing differences between three groups. GraphPad Prism 6.0 (GraphPad Software Inc., La Jolla, CA, USA) was used for all statistical analyses. The results are presented as the mean values ± standard error of the mean (SEM), and *P* < 0.05 was considered statistically significant.

## Result

### Increase of Rspo2-expressing synoviocytes and chondrocytes in RA mice and in patients with RA

**Fig. 1:**
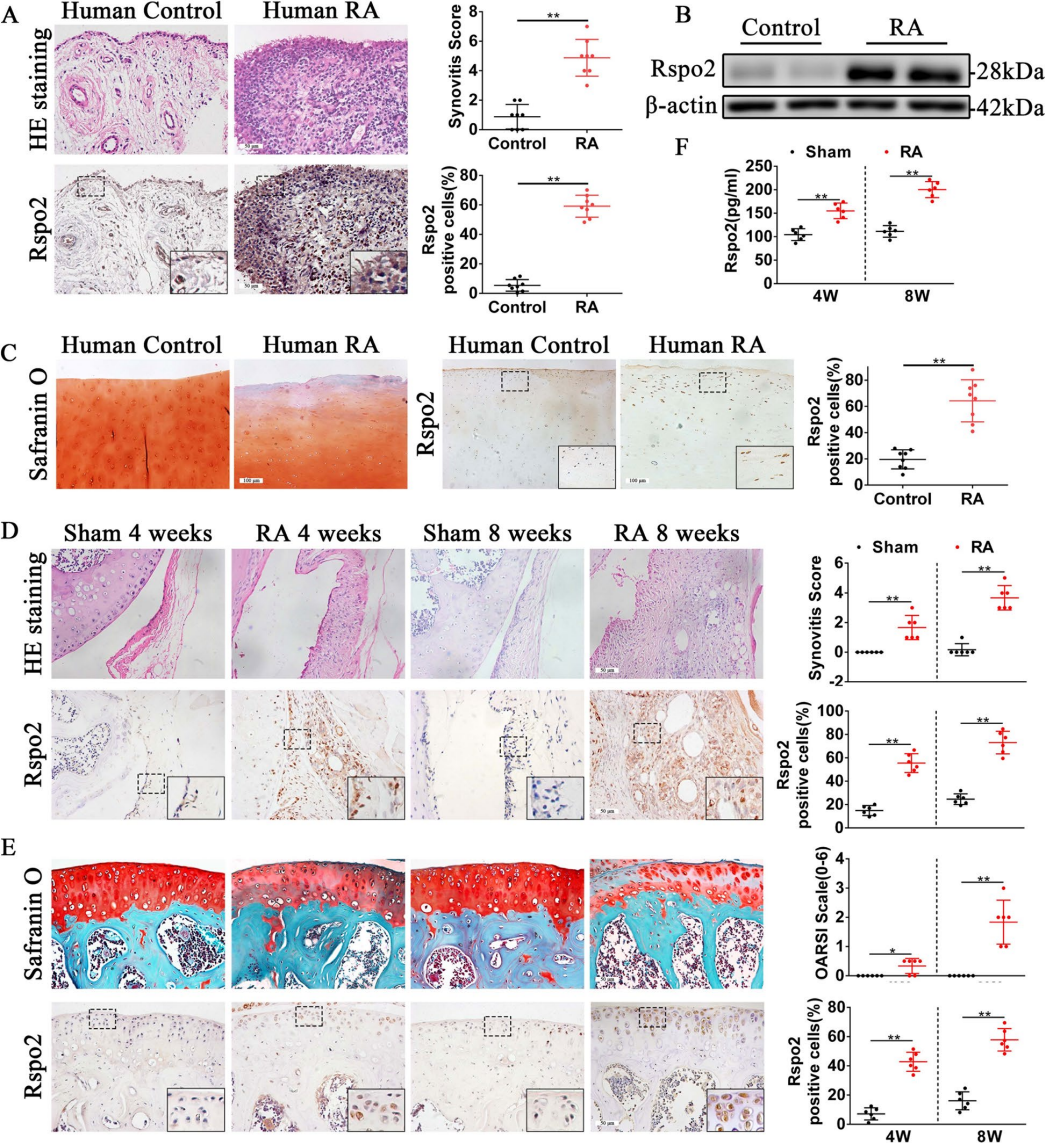
The expression of Rspo2 in RA mouse model and RA patients. **A** Representative images and statistical analysis of HE staining and Rspo2 immunohistochemical staining in control and RA human synovial tissue (*n* = 8 per group). Scale bars: 50 μm. **B** Western blot of Rspo2 expression in human synovial tissue. **C** Representative images and statistical analysis of Safranin O/Fast Green staining and Rspo2 immunohistochemical staining in control and RA human cartilage tissue (*n* = 8 per group). Scale bars: 100 μm. **D** Representative images and statistical analysis of HE staining and Rspo2 immunostaining in the synovium from a RA mouse model and control group (*n* = 6 per group). Scale bars: 50 μm. **E** Representative images and statistical analysis of Safranin O/Fast Green staining and Rspo2 immunostaining in knee cartilage from a RA mouse model and control group (*n* = 6 per group). Scale bars: 50 μm. **F** Rspo2 concentrations assessed by an ELISA in the serum of sham and RA mice at 4 and 8 weeks after AIA modeling (*n* = 6 per group). Student’s *t*-test. ^*^P < 0.05, ^**^P < 0.01. Data are shown as mean ± SEM

To score synovitis in human synovial tissue samples from controls and RA, we used hematoxylin–eosin (HE) staining and immunohistochemical (IHC) staining to detect its pathological features. Compared to controls, significant synovial hyperplasia and plentiful cell infiltration in the synovium of RA patients were evident, with higher synovitis scores (Fig. 1A). Then, to exploit whether Rspo2 plays a function in the development of RA, we further identified the expression of Rspo2 in human RA synovial tissue. The results of IHC staining showed that Rspo2 was elevated in human RA synovial tissue compared to controls (Fig. 1A). Western blot analysis also indicated a remarkably increased expression of Rspo2 in the synovium of RA patients compared to controls (Fig. 1B). Similarly, results from IHC staining revealed the expression of Rspo2 was increased in human RA cartilage tissue, combined with more severe damage (Fig. 1C). We then identified histological features and Rspo2 expression in the synovium and cartilage of mouse RA model, antigen-induced arthritis (AIA), at 4 and 8 weeks. As shown by HE staining, the synovium of RA mice manifested elevated synovitis scores with high level of synovial hyperplasia and plentiful cell infiltration compared to controls, and the IHC staining revealed a significant augment in Rspo2 expression in RA mice (Fig. 1D). We subsequently evaluated the expression of Rspo2 in cartilage in RA mice. Likewise, a minority of Rspo2-positive chondrocytes were found in healthy cartilage, while in RA mice Rspo2 was incrementally upregulated and accompanied by more severe cartilage damage (Fig. 1E). In addition, an enzyme-linked immunosorbent assay (ELISA) determined an apparent increase of Rspo2 content in serum from RA mice (Fig. 1F). In conclusion, these results suggest that Rspo2 expression is elevated both locally and systemically as RA progresses, demonstrating a potential function of Rspo2 in the pathogenesis of RA.

### Rspo2 upregulates the expression of LGR5 and β-catenin in fibroblast-like synoviocytes and chondrocytes

**Fig. 2:**
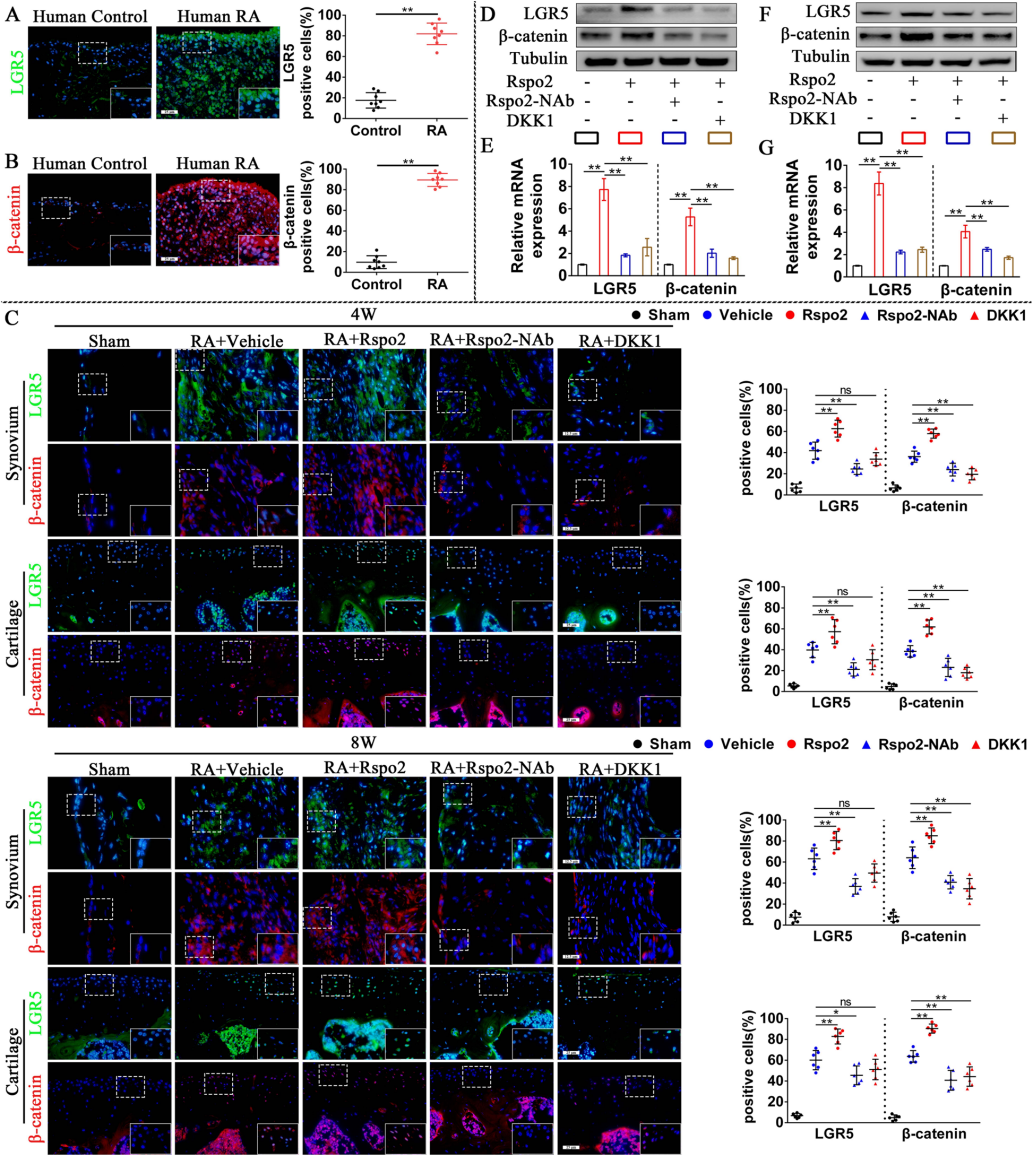
Increased Rspo2 upregulates the expression of LGR5 and β-catenin in fibroblast-like synoviocytes and chondrocytes. **A**,** B** Immunofluorescence staining and statistical analysis of LGR5 (**A**) and β-catenin (**B**) in human synovium (*n* = 8 per group). Scale bar: 25 μm. C Immunofluorescence staining and statistical analysis of LGR5 and β-catenin in the synovium and knee cartilage from sham and RA mice administrated with a vehicle, rmRspo2, Rspo2-NAb, or rmDKK1 for 4 and 8 weeks (*n* = 6 per group). Scale bar: 12.5 μm and 25 μm. **D**,** E** Western blot (**D**) and quantitative PCR analysis (**E**) of LGR5 and β-catenin in rhRspo2-treated FLS with or without Rspo2-NAb or rhDKK1 (*n* = 3 per group). **F**,** G** Western blot (**F**) and quantitative PCR analysis (**G**) of LGR5 and β-catenin in rhRspo2-treated primary chondrocytes with or without Rspo2-NAb or rmDKK1 (*n* = 3 per group). Student’s *t*-test or one-way analysis of variance (ANOVA) and Tukey’s multiple comparison test. ^*^P < 0.05, ^**^P < 0.01; ns, no significance. Data are shown as mean ± SEM

Rspo2 has been identified as an agonist for Wnt/β-catenin signaling to promote chondrocytes hypertrophic differentiation [21], and leucine-rich repeat-containing G-protein-coupled receptor 5 (LGR5), as the ligand for Rspo2, is highly expressed in chondrocytes [22]. However, the role of Rspo2 and LGR5 in the progression of RA, especially in the aggressive phenotype of fibroblast-like synoviocytes (FLS) and chondrocytes homeostasis, had been unclear. Zhang et al. had previously reported that R-spondins might collaborate with LGR5 to regulate the Wnt/β-catenin signaling [23]. Given these findings, we postulated that Rspo2 might activate the Wnt/β-catenin signaling through the upregulation of LGR5. To investigate this hypothesis, we first examined the expression of LGR5 and β-catenin in the RA cartilage and synovium. Results from immunofluorescence (IF) showed that LGR5 and β-catenin were highly expressed in human RA synovial tissue compared to the controls (Fig. 2A, B). To investigate the role of Rspo2 on the expression of LGR5 and β-catenin in vivo, mice after inducing AIA were administrated with recombinant mouse Rspo2 (rm2) or Rspo2 neutralizing antibody (Rspo2-NAb) or rmDKK1 (a potent inhibitor of Wnt signaling pathway). Compared with controls, distinct elevation in the number of LGR5- and β-catenin-positive cells in synovium and articular cartilage were detected in rmRspo2-treated mice both at 4 weeks and at 8 weeks post AIA. Interestingly, downregulated LGR5 and β-catenin were observed in synovium and cartilage of Rspo2-NAb-treated RA mice, while obviously reduced β-catenin-positive cells, not LGR5-, was observed in synovium and cartilage in rmDKK1-treated RA mice (Fig. 2C).

To further investigate the role of Rspo2 on the expression of LGR5 and β-catenin in FLS or primary chondrocytes in vitro, FLS or primary chondrocytes were stimulated with recombinant human Rspo2 (rhRspo2) or rmRspo2, with or without Rspo2-NAb or rh/rmDKK1. FLS treated with rhRspo2 exhibited a significant increase in the protein and mRNA levels of LGR5 and β-catenin, and this impact was effectively ameliorated by Rspo2-NAb (Fig. 2D, E). Additionally, upregulated protein and mRNA expression levels of β-catenin and LGR5 were remarkably reversed by rhDKK1 (Fig. 2D, E). As well, elevated protein and mRNA expression of β-catenin and LGR5 in rmRspo2-treated primary chondrocytes were significantly reduced by Rspo2 neutralizing antibody or rmDKK1 (Fig. 2F, G). These findings suggested that Rspo2 upregulated the protein and mRNA expression of β-catenin and LGR5 in the progression of RA.

### Neutralization of Rspo2 or inhibition of Wnt/β-catenin signaling attenuates synovitis and articular cartilage damage

**Fig. 3:**
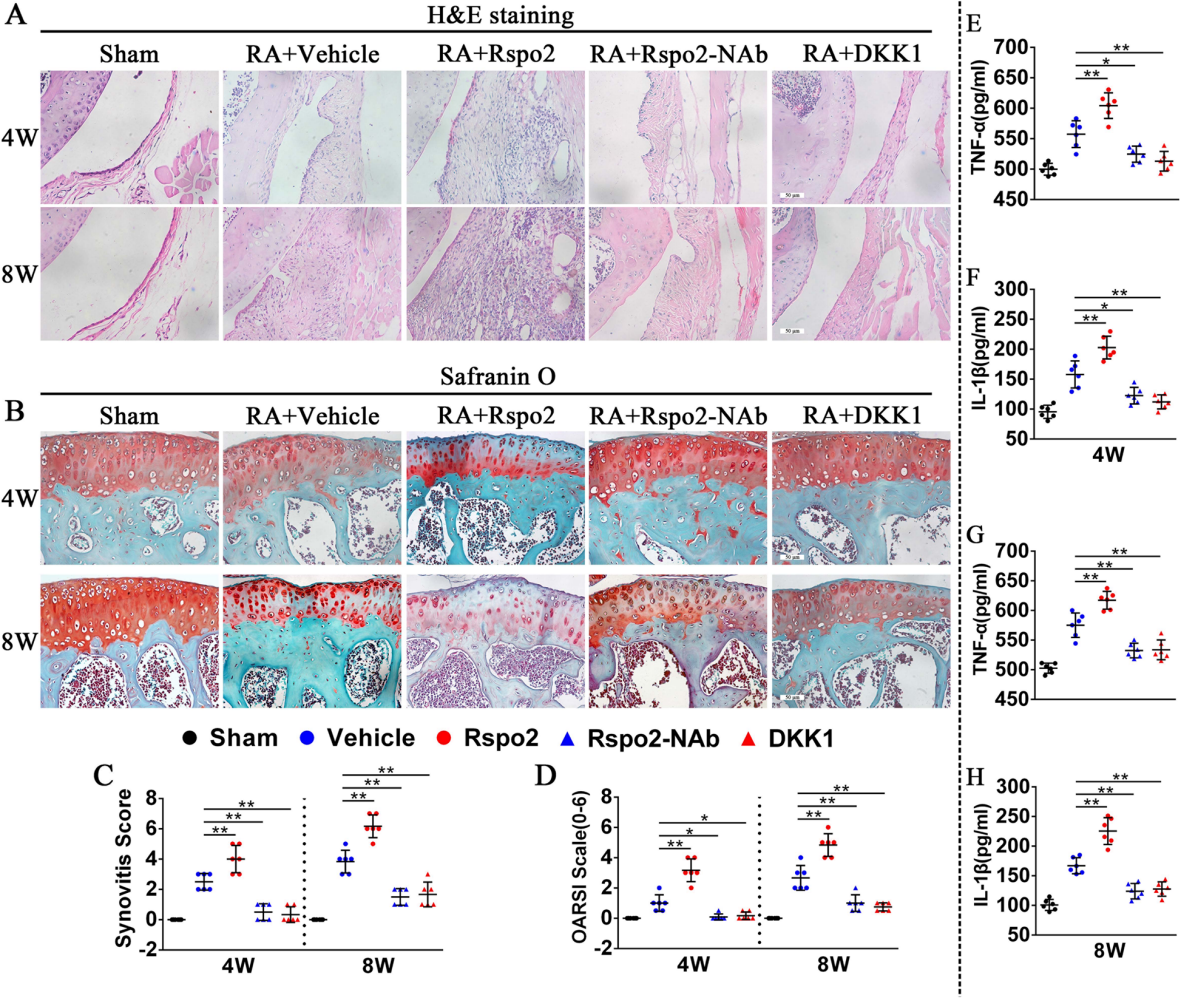
Rspo2 causes strikingly progressive synovial hyperplasia, production of inflammatory cytokines, and articular cartilage loss in RA mice. **A** HE staining of synovial tissues from sham and RA mice administrated with vehicle, rmRspo2, Rspo2-NAb, and rmDKK1 for 4 and 8 weeks. Scale bars: 50 μm. **B** Safranin O/Fast Green staining of knee cartilage from sham and RA mice treated with vehicle, rmRspo2, Rspo2-NAb, and rmDKK1 for 4 and 8 weeks. Scale bars: 50 μm. **C** Synovitis score for the joints described in** A** (*n* = 6 per group). **D** Osteoarthritis Research Society International grades for the joints described in **B** (*n* = 6 per group). **E**–**H** TNF-α and IL-1β concentrations assessed by an ELISA in the serum of sham and RA mice treated with vehicle, rmRspo2, Rspo2-NAb, and rmDKK1 for 4 and 8 weeks (*n* = 6 per group). One-way analysis of variance (ANOVA) and Tukey’s multiple comparison test. ^*^P < 0.05, ^**^P < 0.01. Data are shown as mean ± SEM

To further determine the effect of Rspo2 and β-catenin in the development of RA, 12-week-old male C57BL/6 J mice were administrated by intraarticular injection of rmRspo2, Rspo2-Nab, or rmDKK1 once a week for 4 and 8 weeks after AIA. Compared to controls, an obvious increase of synovitis scores with marked synovial hyperplasia and inflammatory cell infiltration was found in the synovium of RA mice treated with rmRspo2, at both 4 weeks and 8 weeks after AIA, while treatment with Rspo2-NAb or rmDKK1 obviously attenuated synovitis score in RA mice at both 4 weeks and 8 weeks after AIA (Fig. 3A, C). Moreover, rmRspo2 apparently increased OARSI scores compared to controls, manifested by a reduced number of chondrocytes, a diminishing amount of proteoglycans, and worse cartilage erosion at both 4 weeks and 8 weeks post AIA, as evidenced by the OARSI scale. While neutralization of Rspo2 or inhibition of Wnt/β-catenin signaling resulted in an obviously lower OARSI score than controls (Fig. 3B, D). Given that synovitis aggravated after rmRspo2 treatment, we postulated that Rspo2 may affect synovitis via regulation of the inflammatory cytokines. ELISA revealed an increase of TNF-α and IL-1β levels in serum from mice treated with rmRspo2 at both 4 weeks and 8 weeks after AIA. Administration of Rspo2-NAb or rmDKK1 for 4 and 8 weeks effectively lowered TNF-α and IL-1β levels in serum from RA mice after AIA (Fig. 3E–G). Taken together, these data demonstrated that Rspo2 exacerbated articular cartilage damage and synovitis to promote the progression of RA, whereas Rspo2-NAb or rmDKK1 could effectively mitigate the development of RA.

### Rspo2 enhances FLS aggressive phenotype and production of proinflammatory cytokines in RA synovium

**Fig. 4:**
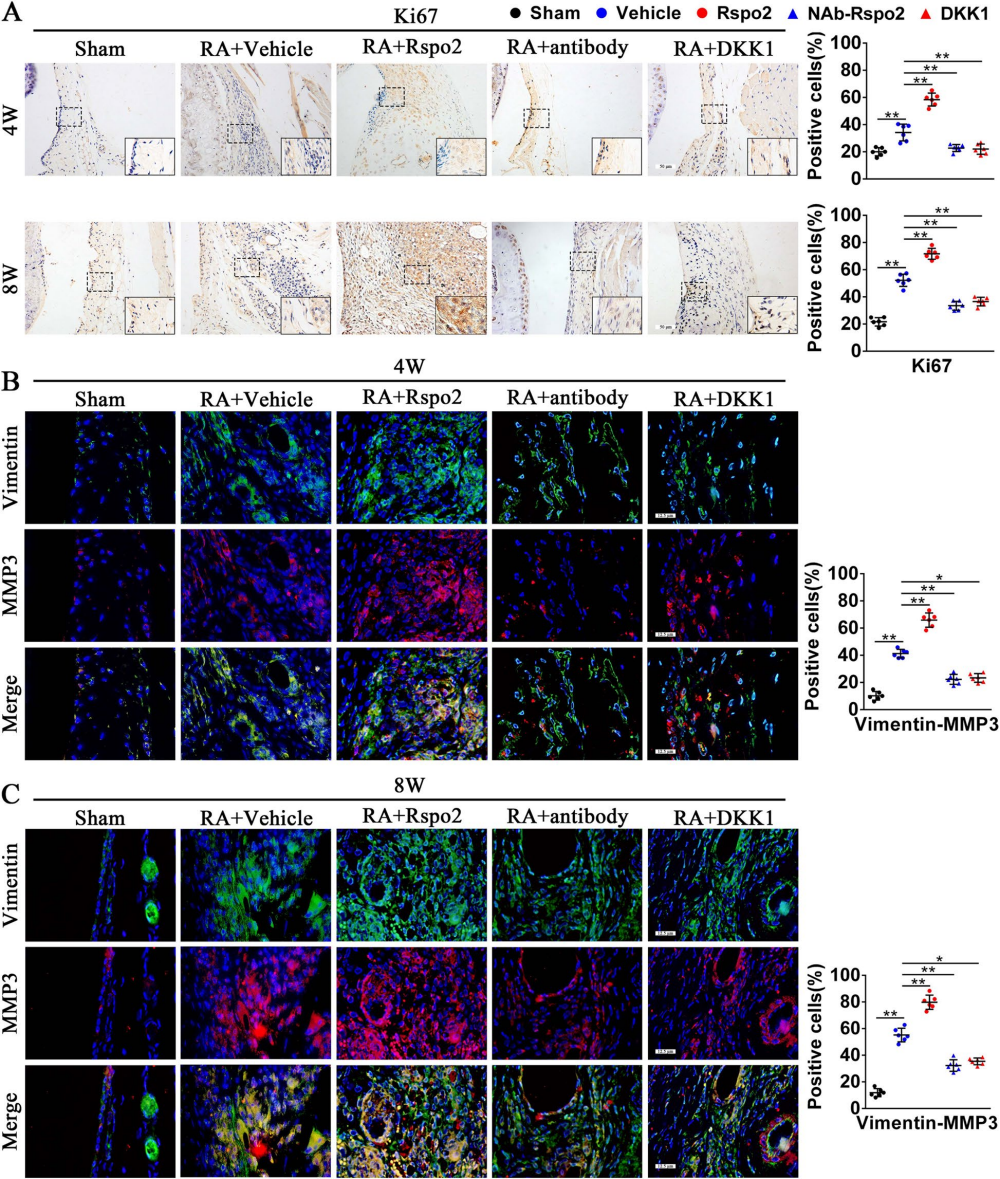
Rspo2 enhances FLS aggressive phenotype in RA synovium. **A** Immunohistochemical staining and statistical analysis of Ki67 in synovial tissues from sham and RA mice treated with vehicle, rmRspo2, Rspo2-NAb, and rmDKK1 for 4 and 8 weeks (*n* = 6 per group). Scale bars: 50 μm. **B**,** C** Immunofluorescence dual staining and statistical analysis of Vimentin and MMP3 in synovial tissues from sham and RA mice treated with vehicle, rmRspo2, Rspo2-NAb, and rmDKK1 for 4 weeks (**B**) and 8 weeks (**C**) (*n* = 6 per group). Scale bars: 12.5 μm. One-way analysis of variance (ANOVA) and Tukey’s multiple comparison test. ^*^P < 0.05, ^**^P < 0.01. Data are shown as mean ± SEM

Synovial hyperplasia and infiltration are well-established crucial driving factors in RA and consist of FLS proliferation, migration, and invasion [20]. The analysis of histomorphometric results after rmRspo2 treatment showed that synovitis progressed over time in RA mice. We then examined the effects of Rspo2 on FLS proliferation and invasion. The results of IHC staining showed that a significant increase of Ki67-positive cells was observed in the synovium of rmRspo2-treated RA mice. However, Rspo2-NAb or rmDKK1 treatment effectively lowered the increase in Ki67-positive cells, which showed decreased proliferation of synoviocytes (Fig. 4A). Additionally, rmRspo2 administration caused significantly elevated expression of MMP3 in RA synovium, along with increased colocalization of Vimentin (an FLS marker) compared to controls, which suggested that Rspo2 could significantly facilitate FLS proliferation and invasion, while the administration of Rspo2-NAb or rmDKK1 caused obviously decreased expression of MMP3 in synovium, as well reduced colocalization of Vimentin compared to controls (Fig. 4B, C), indicating that Rspo2 promoted FLS proliferation and invasion in the progression of RA via activating the β-catenin signaling pathway.

**Fig. 5:**
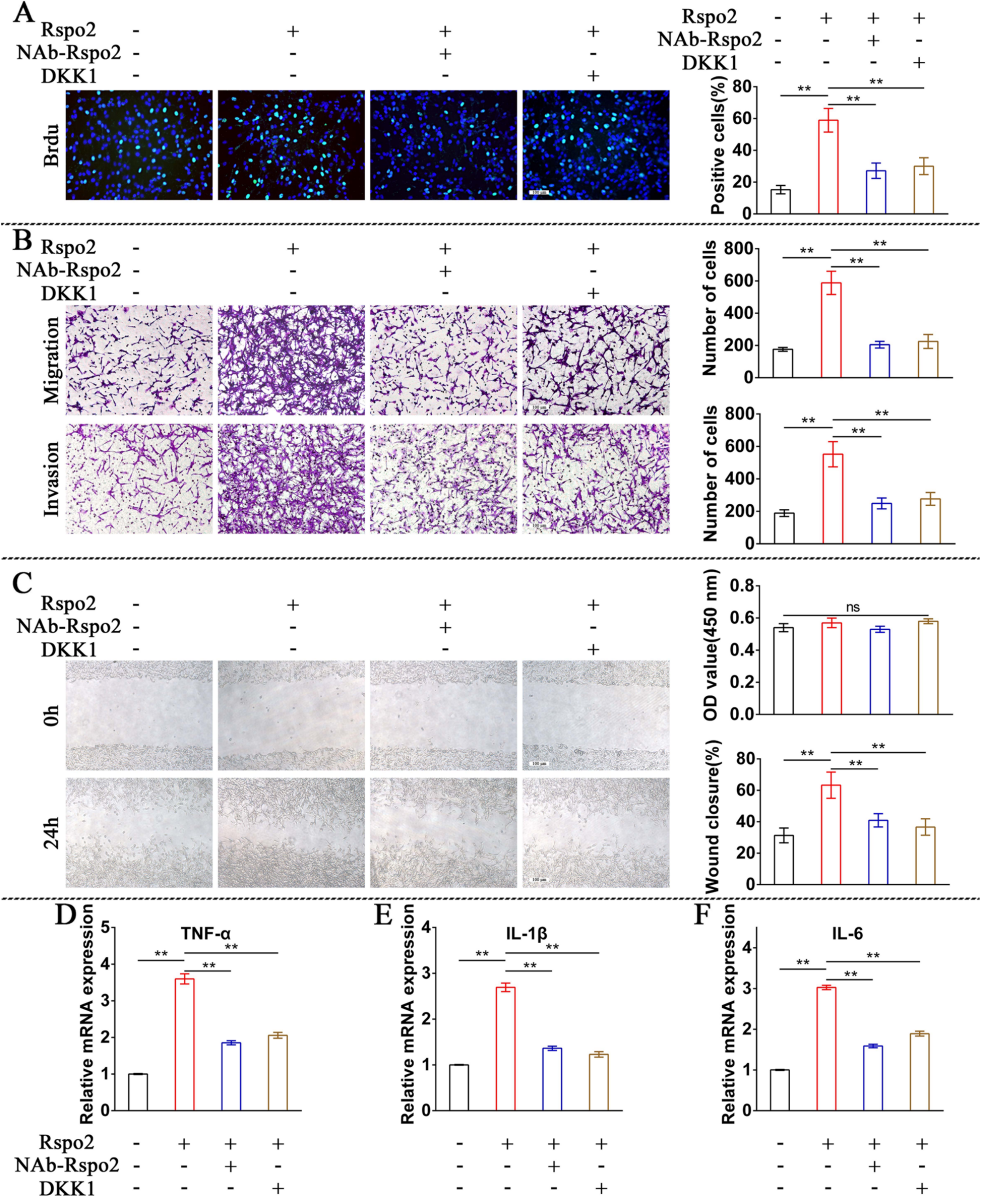
Recombinant Rspo2 promotes proliferation, migration, invasion, and the expression of inflammatory cytokines in FLS. **A** Representative images and quantitative analysis of immunofluorescence staining for BrdU (green) in rhRspo2-treated FLS with or without Rspo2-NAb or rhDKK1 (*n* = 3 per group). Scale bar: 25 μm. **B**, **C** Representative images and quantitative analysis of Transwell assays (**B**) and wound-healing assays (**C**) in rhRspo2-treated FLS with or without Rspo2-NAb or rhDKK1 (*n* = 3 per group). Scale bar: 100 μm. **D**–**F** Relative mRNA expression level of TNF-α (**D**), IL-1β (**E**), and IL-6 (**F**) in rhRspo2-treated FLS with or without Rspo2-NAb or rhDKK1 (*n* = 3 per group). One-way analysis of variance (ANOVA) and Tukey’s multiple comparison test. ^*^P < 0.05, ^**^P < 0.01. Data are shown as mean ± SEM

Next, we attempted to investigate the effects of Rspo2 on FLS proliferation, migration, and invasion. FLS were treated with rhRspo2, rhRspo2 plus Rspo2-NAb, rhRspo2 plus rhDKK1, or the vehicle. The number of BrdU + cells was greatly augmented in FLS treated with rhRspo2 compared with FLS treated with vehicle, and this effect was significantly attenuated by Rspo2-NAb or rhDKK1 (Fig. 5A). These findings indicated that rhRspo2 promoted FLS proliferation through activation of the β-catenin pathway. FLS administrated with rhRspo2 displayed enhanced migratory and invasive abilities, as evidenced by substantially augmented numbers of cells and the improved percentage of wound healing compared with their controls, whereas Rspo2-NAb or rhDKK1 effectively suppressed the migratory and invasive abilities of FLS triggered by rhRspo2 (Fig. 5B, C). In addition, Rspo2-NAb or rhDKK1 significantly reduced the inflammatory cytokine production, including TNF-α, IL-1β, and IL-6, by FLS stimulated by rhRSPO2 (Fig. 5D–F). Interestingly, our data showed that recombinant human LGR5 (rhLGR5) did not significantly promote FLS migration and invasion, or the upregulation of inflammatory cytokines from FLS, such as MMP3, TNF-α, IL-1β, and IL-6 (Fig. S1A-C). These findings demonstrated that LGR5 might not directly regulate the aggressive phenotype of FLS and chondrocyte homeostasis. Given our evidence identified that the expression of LGR5 was markedly upregulated by Rspo2 in the synovium of human patients and mice with RA (Fig. 2A, C, D, E). Furthermore, Zhang et al. had previously reported that R-spondins might collaborate with LGR5 to regulate the Wnt/β-catenin signaling [23]. Thus, we suspected that LGR5 might be involved in regulating aggressive phenotype of FLS and chondrocyte homeostasis by functioning as the ligand for Rspo2. To investigate this hypothesis, siRNA targeting LGR5 was transfected into FLS (Fig. S2A, B). We found that the enhanced migratory and invasive abilities (Fig. S2C, D), as well as upregulated MMP3, TNF-α, IL-1β, and IL-6 (Fig. S2E) in FLS induced by Rspo2, were alleviated by LGR5 knockdown. These data suggested that the effect of Rspo2 on the aggressive phenotype of FLS could be ameliorated by LGR5 knockdown. These findings demonstrated that Rspo2 promoted FLS migration, invasion, and inflammatory cytokine production through upregulating LGR5 and activating the Wnt/β-catenin signaling pathway.

### Rspo2 disrupts chondrocyte homeostasis in the development of RA

**Fig. 6:**
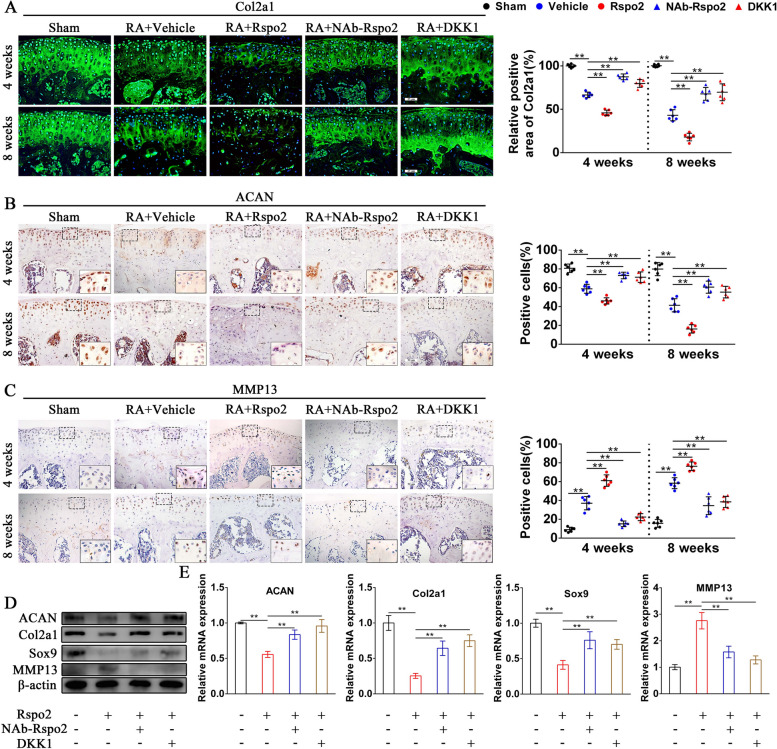
Rspo2 disrupt chondrocyte homeostasis in RA. **A** Immunofluorescence staining and statistical analysis of Col2a1 in the knee cartilage from sham and RA mice administered with a vehicle, rmRspo2, Rspo2-NAb, or rmDKK1 (*n* = 6 per group). Scale bar: 25 μm. **B**,** C** Representative immunohistochemical images and statistical analysis of ACAN and MMP13 in the knee cartilage from sham and RA mice administrated with a vehicle, rmRspo2, Rspo2-NAb, or rmDKK1 (*n* = 6 per group). Scale bar: 25 μm. **D**,** E** Western blot (**D**) and quantitative PCR analysis (**E**) of ANAN, Col2a1, Sox9, and MMP13 in rmRspo2-treated chondrocytes with or without Rspo2-NAb or rmDKK1 (*n* = 3 per group). One-way analysis of variance (ANOVA) and Tukey’s multiple comparison test. ^*^P < 0.05, ^**^P < 0.01. Data are shown as mean ± SEM

In the progression of RA, cartilage damage is a crucial pathological feature. We then investigate the effect of Rspo2 on articular chondrocyte homeostasis during RA development. Significantly decreased expression of type 2 collagen (Col2a1) and aggrecan (ACAN) and increased expression of matrix metalloproteinase 13 (MMP13) were found in knee joint cartilage of rmRspo2-treated mice. Strikingly, Rspo2-NAb or rmDKK1 treatment effectively improved chondrocyte homeostasis by reversing the decrease of Col2a1 and ACAN as well as the increase in MMP13 expression (Fig. 6A–C). Western blot analysis and RT-qPCR results indicated that ACAN, Col2a1, and Sox9 protein and mRNA production were downregulated, whereas MMP13 protein and mRNA production were upregulated in mouse primary chondrocytes after treatment with rmRspo2. These impacts were significantly reversed by Rspo2-NAb or rmDKK1 (Fig. 6D, E). Although the expression of LGR5 was obviously upregulated in chondrocytes triggered by Rspo2 (Fig. 2C, F, G), the results from western blot and qPCR suggested that chondrocytes homeostasis could not be obviously disrupted by treating with rhLGR5 (Fig. S1D, E). Taken together, these findings demonstrated that Rspo2 suppressed catabolism and enhanced anabolism to regulate chondrocyte homeostasis by activating the Wnt/β-catenin signaling pathway.

## Discussion

Due to the increasing insights into the pathogenesis of RA, many effective drugs are now available for the treatment of RA, among which the use of biologics and targeted synthetic disease-modifying antirheumatic drugs (tsDMARDs) is considered a landmark in the treatment of RA [24]. As a result of the high cost and increased risk of serious infections and skin cancer, biologic agents and tsDMARDs are limited in clinical therapy [25]. Thus, an in-depth insight into the pathogenesis of RA is beneficial to provide a potentially novel therapeutic target. A recent study has demonstrated targeting RA FLS is more vital for RA therapy than applying tsDMARDs [25]. By migrating and invading with tumor-like properties, RA FLS exhibit pivotal effects on synovitis, cartilage damage, and subsequent bone disruption [25]. In this study, we demonstrated that Rspo2 is a vital factor promoting the aggressive phenotype and the production of inflammatory cytokines by FLS, as well as disturbing chondrocytes homeostasis during the pathogenesis of RA. The upregulation of Rspo2 was shown to facilitate synovitis and cartilage damage to exacerbate the progression of experimental RA primarily through the activation of Wnt/β-catenin signaling pathway. Our findings identified Rspo2 targeting FLS aggressive phenotype and chondrocytes homeostasis as a possibly novel therapy for RA treatment.

RA is characterized by an invasive synovial hyperplasia, which eventually causes cartilage and bone degeneration. A markedly elevated FLS in RA joint contribute to the transformation of the synovial lining from a fragile structure into an aggressive hyperplastic pannus. FLS are a crucial component of the aggressive synovium implicated in the initiation and maintenance of destructive joint inflammation. Therefore, targeting inhibition of aberrant FLS proliferation might be a potential approach to attenuate RA. Our findings showed that Rspo2 significantly increased BrdU-positive cells in FLS in vitro. Moreover, elevated Ki-67-positive cells were further observed in synovium of AIA mice treated with rmRspo2. Wang et al. previously reported that knockdown of Rspo2 significantly inhibits nasopharyngeal carcinoma cell lines SUNE-6-10B and CNE-1 cell proliferation [26]. Another essential finding regarding Rspo2 was that exogenous Rspo2 or overexpression of Rspo2 promoted proliferation in tongue squamous cell carcinoma [27]. One study showed that enhanced transcription of RSPO2 further promoted the proliferation of granulosa cells [28]. Consistent with these findings, our data suggest Rspo2 enhances FLS proliferation, which might be associated with RA pathogenesis.

Another pivotal pathologic feature of RA FLS is an aggressive phenotype facilitating joint destruction. Besides promoting the proliferation of FLS, Rspo2 also plays an important effect in migration and invasion of FLS. Suppression of Rspo2 expression levels by siRNA significantly inhibited cell migration and invasion in gastric cancer cell lines (AGS and BGC-823) [23]. Moreover, knockdown of Rspo2 obviously suppressed migration and invasion of SUNE-6-10B and CNE-1 cells [26]. However, whether Rspo2 regulated these phenotypes in FLS during RA progression was still unclear. Our results provided experimental evidence that Rspo2 could activate FLS aggressive phenotype, as evident by enhanced migration and invasion of FLS treated with rhRspo2 in vitro. The activation and augment of RA FLS are associated with the disease duration, the extent of synovial macrophage infiltration, and the progression of cartilage erosion [29, 30]. Studies in mice also showed that activation of FLS is essential for driving arthritis in TNF transgenic mice and collagen-induced arthritis mice [31, 32]. At the interface between synovium and cartilage in the rheumatoid joint, RA FLS upregulating the expression of MMPs, such as MMP1, MMP3, and MMP13, destruct the ECM of the joint tissues and enhance FLS invasion [3]. Smolen et al. have showed that the upregulated expression of MMPs has been found in the synovial intimal lining layer in RA patient with the occurrence of symptoms less than 1 week [33]. The above results highlighted the important role of the RA FLS aggressive phenotype and its upregulated MMPs in RA progression. Similar to these findings, we also indicated that recombinant Rspo2 could significantly promote synovial hyperplasia and activate FLS by upregulating the expression of MMP3 and Vimentin in the synovium of RA mouse model. Taken together, the abovementioned data suggested that Rspo2 might promote the aggressive phenotype of FLS to aggravate RA progression by increasing the expression of MMP3.

The last important pathologic feature of RA FLS is the increased production of proinflammatory cytokines, chemokines, and proangiogenic factors, involved in the progression of RA. Our previous study had indicated that increased proinflammatory cytokines of FLS might aggravate the development of RA [20]. Cytokine inhibitors have distinctly showed a crucial effect of TNF-α and IL-6 in disease pathogenesis [34]. These findings reveal a powerful association between the pro-inflammatory cytokines of FLS and the severity of RA, but the modulating role of Rspo2 on the production of proinflammatory cytokines in RA FLS remains obscure. Our findings demonstrated that recombinant Rspo2 promoted the production of proinflammatory cytokines (such as TNF-α, IL-1β, and IL-6) by FLS in vitro. In addition, more severe synovitis was observed in the RA mouse model after administration of rmRspo2. Previous studies had demonstrated that Rspo2 could mitigate inflammation response in IL-1β-treated chondrocytes [18] and regulate inflammation response in acute ozone-induced lung injury [35]. Consistent with previous results, our findings indicated that rmRspo2 aggravated synovitis by promoting the production of proinflammatory cytokines by FLS in an RA mouse model.

FLS exert a vital function of destructing RA joint structures, including cartilage and support soft tissue structure, by their unique aggressive feature and the production of plentiful amounts of proteases (such as MMP3 and MMP13) [3]. In our study, we identified that recombinant Rspo2 remarkably upregulated the expression of MMP3 in FLS in RA mice, which suggested that Rspo2 might contribute to cartilage destruction by the elevated production of MMP3 in FLS. Our previous study had demonstrated that Rspo2 secreted by synovium could disrupt cartilage homeostasis during osteoarthritis progression [22]. However, whether Rspo2 exacerbates articular cartilage degeneration in RA is still unclear. Our data showed that recombinant Rspo2 promoted catabolism and inhibited anabolism in primary mouse chondrocytes. Importantly, rmRspo2-treated RA mice exhibited more severe cartilage destruction in knee joint. Knights et al. had reported that Prg4^hi^ synovial lining fibroblasts secreted Rspo2 that might trigger pathological joint crosstalk during post-traumatic osteoarthritis in mice [36]. These data indicated Rspo2 aggravated articular cartilage degeneration by directly disrupting chondrocyte homeostasis and by upregulating the MMP3 expression level of FLS in RA.

Emerging evidence is illuminating the indispensable regulatory role of Wnt/β-catenin signaling pathway in RA progression [37]. The activation of Wnt/β-catenin signaling pathway can facilitate synovial cell proliferation, promote synovial hyperplasia, cause cartilage degeneration, and destroy joint function in RA [38]. Therefore, investigating the mechanism of the Wnt/β-catenin signaling pathway in regulating the FLS and chondrocyte pathways is of great significance for RA therapy. For the first time, we confirmed that LGR5, a receptor of Rspo2, was distinctly upregulated in the knee joint synovium of RA patients and RA mice. Recombinant Rspo2 could significantly upregulate the expression of LGR5 in FLS and primary mouse chondrocytes, as well as in an RA mouse model. The effects of Rspo2 might be effectively inhibited by Rspo2 neutralization, not recombinant DKK1. Moreover, the decreased expression of LGR5 and β-catenin were observed in human gastric cancer by silencing Rspo2 [23]. We also found that recombinant Rspo2 obviously upregulated the expression of β-catenin in FLS and primary mouse chondrocytes, as well as in an RA mouse model. The effects of Rspo2 could be effectively suppressed by Rspo2 neutralizing antibody or recombinant DKK1. These findings suggest that Rspo2 may exert a main function in the activation of the Wnt/β-catenin pathway in RA. Yet, its specific role in RA remains unclear. Our experimental results revealed that Rspo2-NAb- or rmDKK1-treated RA mice exhibited inhibiting synovitis and attenuating articular cartilage degeneration. It had been reported that Wnt signaling regulated the expression of matrix metalloproteinases (MMPs) in osteoarthritis and lupus nephritis [39, 40]. Like these findings, we also found that Rspo2 neutralization and β-catenin inhibition could obviously reduce the expression of MMP3 in FLS to attenuate the aggressive phenotype of FLS. Additionally, Rspo2 neutralization and recombinant DKK1 both significantly reversed the aggressive phenotypes of FLS and restored the disrupted homeostasis of chondrocyte triggered by recombinant Rspo2 in vitro. To wit, Rspo2 neutralization and recombinant DKK1 could effectively ameliorate the proliferative capacity, migratory capacity, invasive capacity, and proinflammatory cytokines production in FLS, as well as inhibit catabolism and enhance anabolism in chondrocyte homeostasis, which might be implicated in the onset and progression of RA. Furthermore, apart from activating the Wnt signaling, Rspo2 promoted cellular proliferation via the FAK/Src signaling and functioned as an antagonist of the FGF signaling during growth and development [41, 42]. These findings implied that Rspo2 potentially possesses additional roles and regulatory mechanisms in the initiation and progression of RA. As mentioned above, these results suggest that Rspo2 aggravates the progression of RA primarily through the activation of Wnt/β-catenin pathway. Additionally, systemic application of Wnt inhibitors might have unwanted side effects or even be toxic to normal tissues, including increased risk of osteoporosis and cardiovascular diseases [43, 44]. Hence, Rspo2 neutralizing antibody might possess certain advantages over Wnt antagonists as potential therapeutic targets for the treatment of RA.

The key role and underlying mechanism of Rspo2 in RA have been elucidated, but several limitations have yet to be illuminated. As a first limitation, rmRspo2, Rspo2-NAb, or rmDKK1 was injected intra-articularly rather than using transgenic mice. Secondly, although no significant difference in the number of LGR5-positive cells was detected in synovium and articular cartilage between rmDKK1-treated RA mice and their control, elevated protein and mRNA expression of LGR5 in rmRspo2-treated FLS and primary chondrocytes were significantly reduced by Rspo2-NAb or recombinant DKK1. We speculate it is due to the expression of LGR5 in synovium and articular cartilage is regulated not only by Rspo2 or Wnt/β-catenin pathway, but also by other potential mechanisms in vivo. Finally, the R-spondin family consists of four secreted proteins (Rspo1–4) with 60% overall sequence homology and two furin-like cysteine-rich domains [10, 11], and its receptors including LGR4, 5, and 6 [13]. In this study, we just demonstrated the critical role and potential mechanism of Rspo2 and LGR5 in RA development; further studies are needed to explore the effects of R-spondin family and its receptors in the progression of RA.

## Conclusions

In summary, we identified that upregulation of Rspo2 facilitated FLS aggressive phenotype and disrupted chondrocytes homeostasis primarily by Wnt/β-catenin pathway in RA. Importantly, Rspo2 neutralization or Wnt/β-catenin inhibition effectively inhibited synovial inflammation, synovial hyperplasia, and cartilage degradation to attenuate the progression of RA. Hence, our study suggested that targeting Rspo2 neutralizing antibody or recombinant DKK1 by intraarticular supplementation represents a potentially novel therapeutic approach for RA therapy.

### Supplementary Information

**Additional file 1:** **Figure S1.** The role of recombinant LGR5 on aggressive phenotype of FLS and chondrocytes homeostasis. **Figure S2.** LGR5 knockdown attenuates the effect of Rspo2 on the aggressive phenotype of FLS. **Figure S3.** Raw data of western blot images.

## Data Availability

Not applicable.

## Abbreviations

Rspo2: R-spondin-2
RA: Rheumatoid arthritis
AIA: Antigen-induced arthritis
rmRspo2: Recombinant mouse Rspo2
rmDKK1: Recombinant mouse DKK1
rhRspo2: Recombinant human Rspo2
rhDKK1: Recombinant human DKK1
Rspo2-NAb: Rspo2 neutralizing antibody
FLS: Fibroblast-like synoviocytes
LGRs: Leucine-rich repeat-containing G-protein-coupled receptors
LGR5: Leucine-rich repeat-containing G-protein-coupled receptor 5
ECM: Extracellular matrix
HE: Hematoxylin-eosin
IHC: Immunohistochemical
ELISA: Enzyme-linked immunosorbent assay
IF: Immunofluorescence
Col2a1: Type 2 collagen
ACAN: Aggrecan
MMP13: Matrix metalloproteinase 13
tsDMARDs: Targeted synthetic disease-modifying antirheumatic drugs
