# Supplementary material for: Rspo2 exacerbates rheumatoid arthritis by targeting aggressive phenotype of fibroblast-like synoviocytes and disrupting chondrocyte homeostasis via Wnt/β-catenin pathway
Source: Arthritis Res Ther. 2023 Nov 9;25:217. doi: 10.1186/s13075-023-03198-1 (PMC10634117; doi:10.1186/s13075-023-03198-1)
Supplement: Supplementary file 1 — Additional file 1: Figure S1. The role of recombinant LGR5 on aggressive phenotype of FLS and chondrocytes homeostasis. Figure S2. LGR5 knockdown attenuates the effect of Rspo2 on the aggressive phenotype of FLS. Figure S3. Raw data of western blot images. [file 13075_2023_3198_MOESM1_ESM.docx]

**Rspo2 exacerbates rheumatoid arthritis by targeting** **aggressive phenotype of fibroblast-like synoviocytes and disrupting chondrocyte homeostasis via** **Wnt/β-catenin pathway**

Dong Guo^a,c^, Haoyan Pan ^a,c^, Xueying Lu^d^, Zhong Chen^e^, Laixi Zhou^b^, Shuxin Chen^b^, Jin Huang^b^, Xinzhi Liang^a,c^, Zhisheng Xiao^a,c^, Hua Zeng^a,c^, Yan Shao^a,c^, Weizhong Qi ^a,c*^, Denghui Xie^a,c*^, Chuangxin Lin^b*^

**Supplementary figure:**


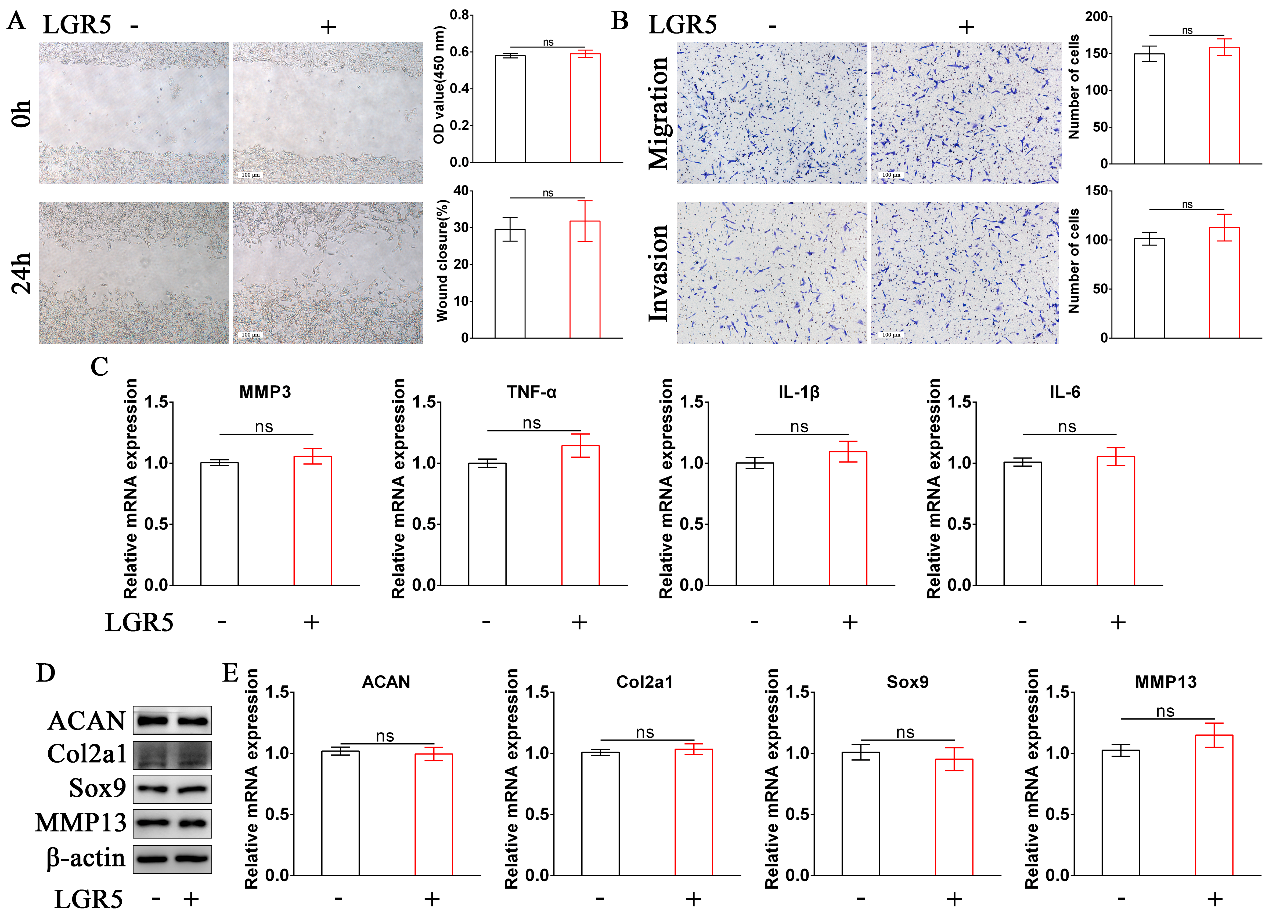
 **Figure S1.** The role of recombinant LGR5 on aggressive phenotype of FLS and chondrocytes homeostasis. **(A, B)** Representative images and quantitative analysis of wound-healing assays (A) and Transwell assays (B) in rhLGR5-treated FLS (n = 3 per group). Scale bar: 100 µm. (**C**) Relative mRNA expression level of MMP3, TNF-α, IL-1β and IL-6 in rhLGR5-treated FLS (n = 3 per group). **(D, E)** Western blot (D) and quantitative PCR analysis (E) of ANAN, Col2a1, Sox9 and MMP13 in chondrocytes treated with recombinant LGR5 (n = 3 per group). Student’s *t*-test. ns, no significance. Data are shown as mean ± SEM.


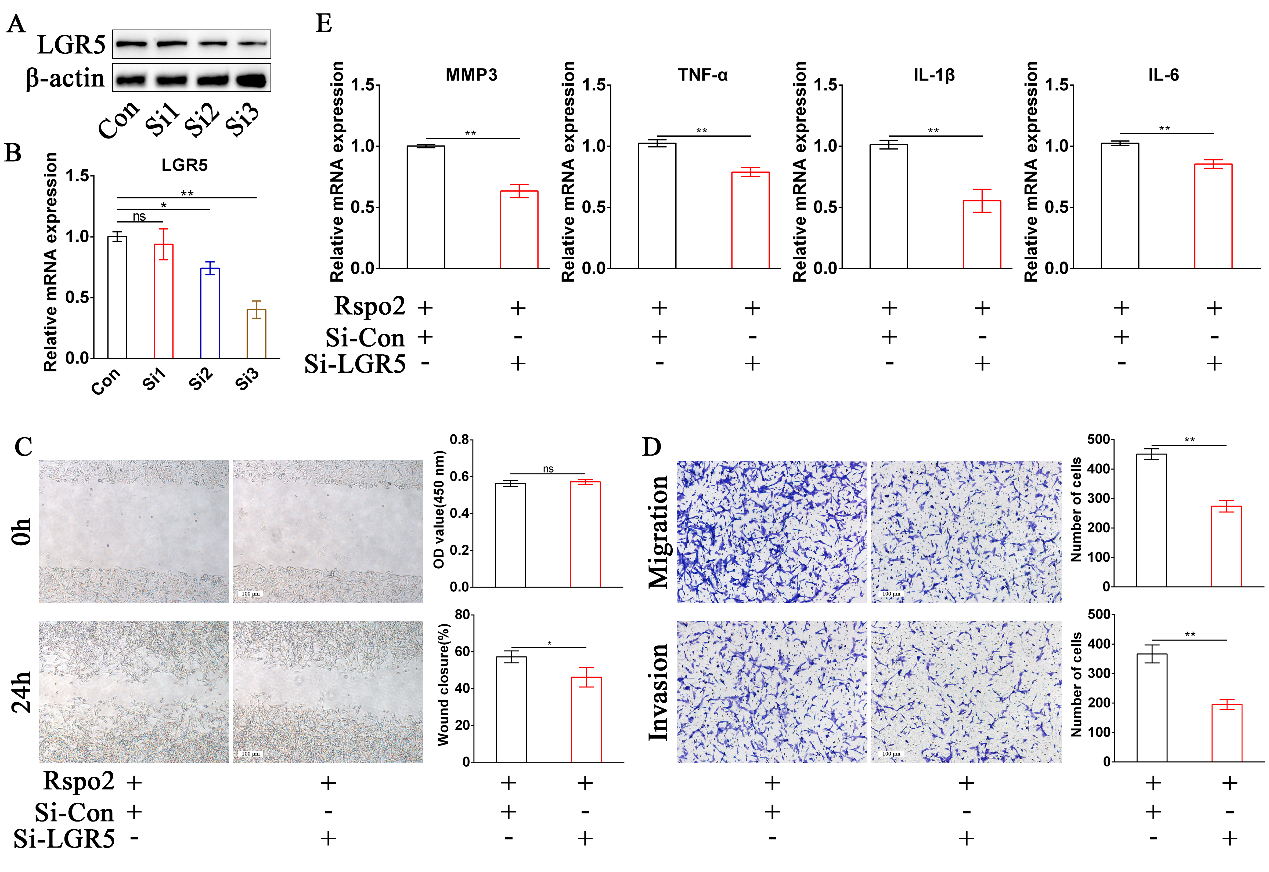
**Figure S2.** LGR5 knockdown attenuates the effect of Rspo2 on the aggressive phenotype of FLS. (**A, B**) Quantitative PCR analysis (A) and western blot (B) of LGR5 in FLS transfected with siRNA targeting LGR5. **(C, D)** Representative images and quantitative analysis of wound-healing assays (C) and Transwell assays (D) in rhLGR5-treated FLS transfected with siRNA-LGR5 or siRNA-control (n = 3 per group). Scale bar: 100 µm. (**E**) Relative mRNA expression level of MMP3, TNF-α, IL-1β and IL-6 in rhLGR5-treated FLS transfected with siRNA-LGR5 or siRNA-control (n = 3 per group). Student’s *t*-test or one-way analysis of variance (ANOVA) and Tukey’s multiple comparison test. *P < 0.05, **P < 0.01; ns, no significance. Data are shown as mean ± SEM.


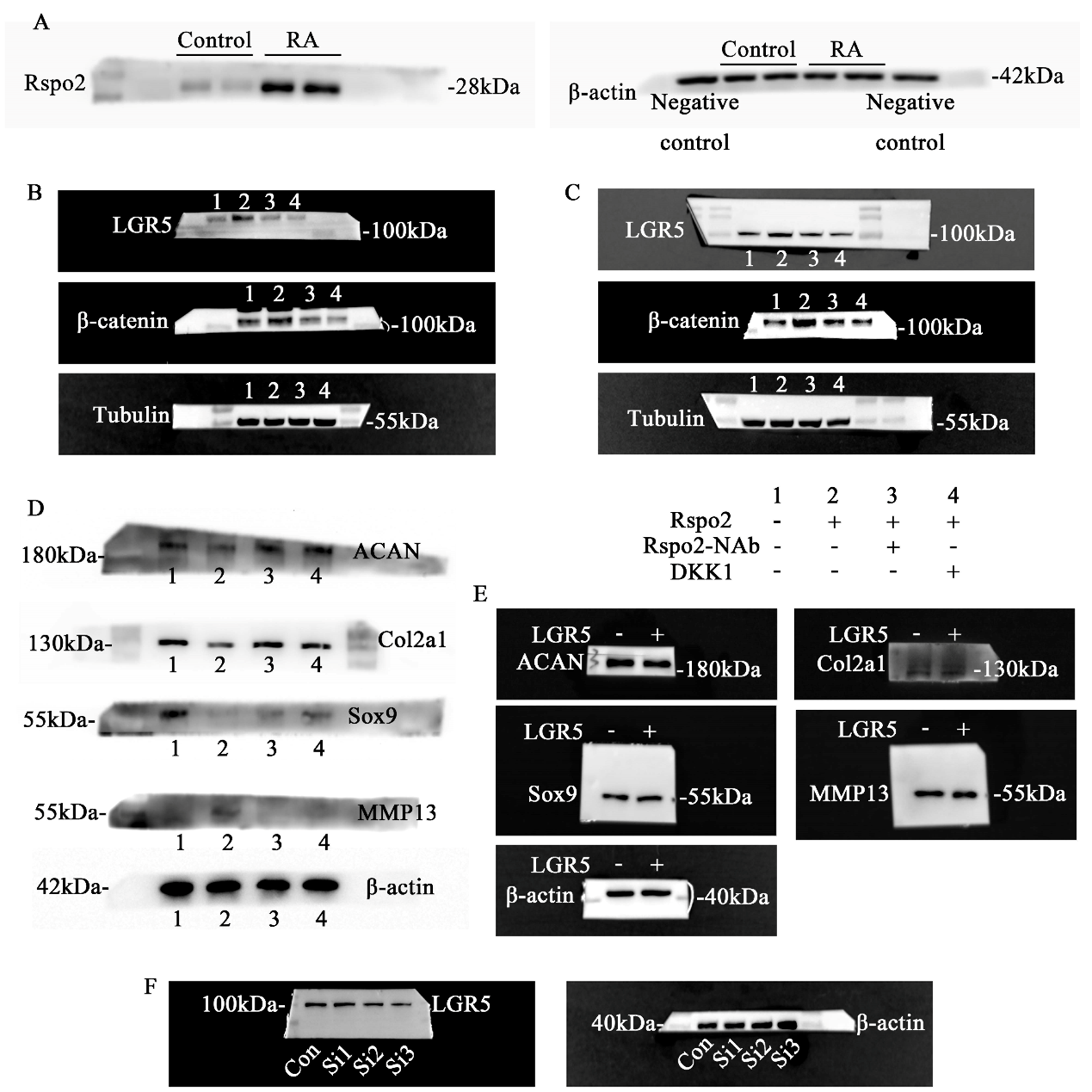
**Figure S3.** Raw data of western blot images. (A) figure 1B. (B) figure 2D. (C) figure 2F. (D) figure 6D. (E) figure S1D. (F) figure S2A.
